# Metastatic Clear Renal Cell Carcinoma in Bladder Following Radical Nephrectomy, Metastasectomy, and Targeted Therapy: A Rare Case Report

**DOI:** 10.1002/ccr3.73557

**Published:** 2026-09-20

**Authors:** Xiaohong Zheng, Peng Jiang, Jiatong Zhou

**Affiliations:** ^1^ Department of Urology The First Affiliated Hospital, Zhejiang University School of Medicine Hangzhou P.R. China

**Keywords:** bladder metastasis, case report, nephrectomy, renal cell carcinoma

## Abstract

Bladder metastasis from renal cell carcinoma (RCC) is relatively uncommon and may occur synchronously or metachronously, potentially without clinical symptoms. Notably, cases of bladder metastasis in advanced RCC following targeted therapy were particularly rare. Here we present the case of a 50‐year‐old female patient who underwent radical left nephrectomy for a renal tumor. Postoperative pathology confirmed clear cell renal cell carcinoma (ccRCC). Following the development of contralateral adrenal and metacarpal metastases, our institution performed metastatic lesion resection, after which the patient received adjuvant sunitinib therapy. Follow‐up PET/CT revealed additional cranial bone metastasis and a suspicious bladder wall lesion. Subsequent surgical interventions included resection of the cranial metastasis and bladder mass, with pathology confirming metastatic ccRCC. Postoperatively, the patient was treated with axitinib combined with toripalimab. Despite treatment, the disease progressed with new widespread skeletal metastases, though no bladder recurrence was observed.

## Key Clinical Message

Bladder metastasis of RCC may occur via hematogenous, lymphatic, or direct spread, though the exact mechanisms remain unclear. Particularly in advanced RCC cases, metastases to uncommon organ sites can be challenging to detect, necessitating thorough clinical evaluation by physicians.

## 1 Introduction

Renal cell carcinoma (RCC) is the 7th most common malignant tumor in men and the 10th most common in women worldwide [1]. Approximately 20%–30% of RCC patients are diagnosed with distant metastasis at the time of initial diagnosis. The most common metastatic sites of RCC include regional lymph nodes (40%–60%), lung (50%–60%), bone (30%–40%), and liver (30%–40%) [2]. With the application of targeted therapy and immunotherapy for RCC [3], the survival time of some patients has been significantly prolonged. This may lead to increased detection of metastases in rare sites, including the esophagus, eyeball, and bladder [4, 5]. Clear cell carcinoma is the most common histological subtype of RCC [6]. Since bladder metastasis of RCC is exceedingly rare, with fewer than 70 reported cases in the literature, we present a rare case of a 50‐year‐old female diagnosed with advanced clear cell RCC who developed multiple bone metastases and subsequent bladder metastasis over more than 2 years of follow‐up.

## 2 Case History/Examination

**FIGURE 1:**
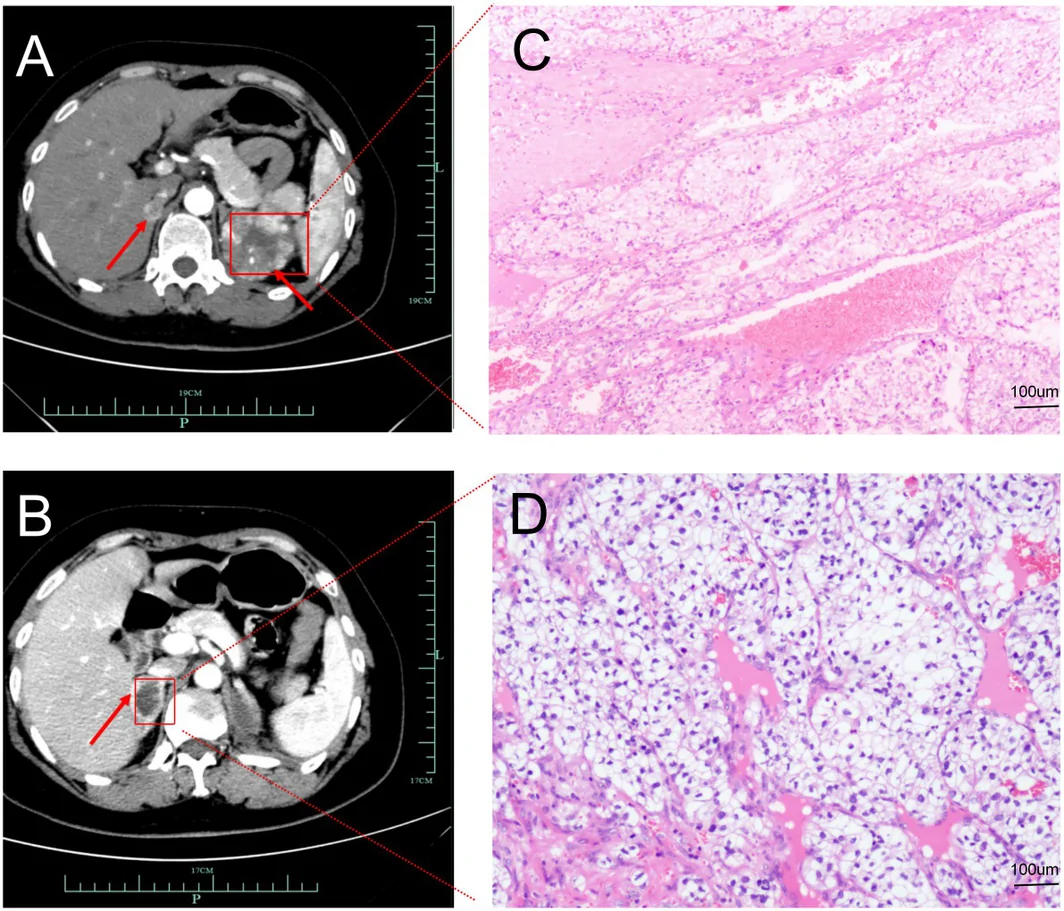
(A) The contrast‐enhanced CT scan demonstrated a mass lesion in the left kidney with metastatic tumor in the right adrenal gland, accompanied by H&E‐staining of left kidney tumor (C). (B) The contrast‐enhanced CT scan demonstrated progression of the metastatic tumor in the right adrenal gland, accompanied by H&E‐staining of metastatic tumor in right adrenal gland (D).

**FIGURE 2:**
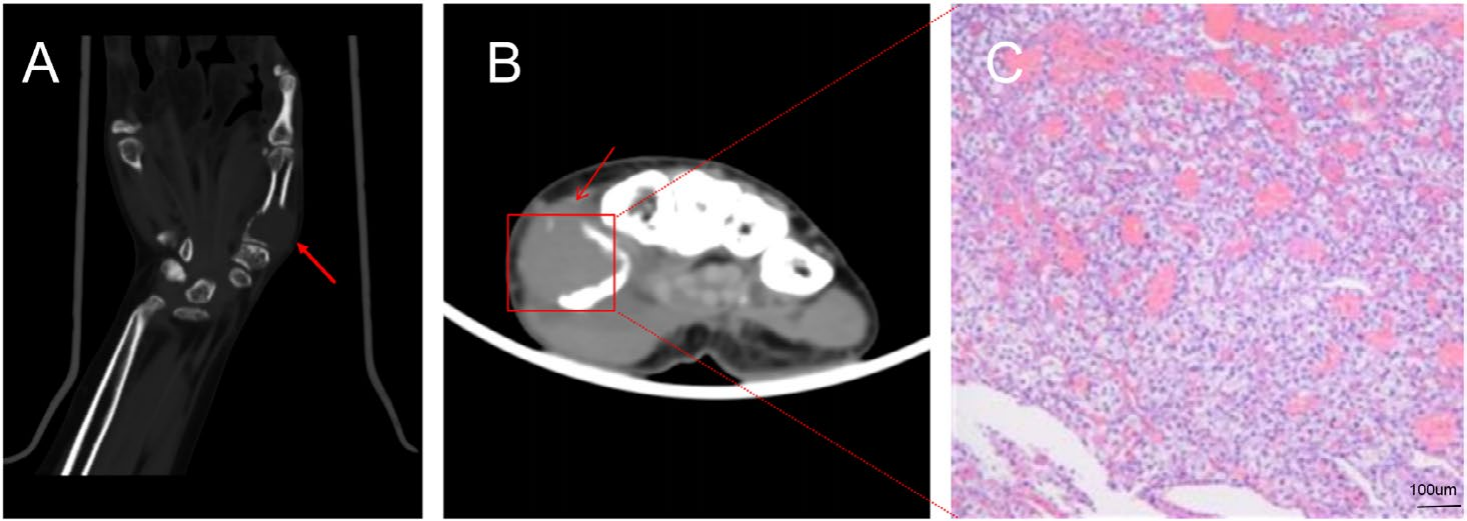
(A, B) The CT scan revealed an osteolytic lesion in the right first metacarpal bone, accompanied by H&E‐staining of the metastatic lesion (C).

**FIGURE 3:**
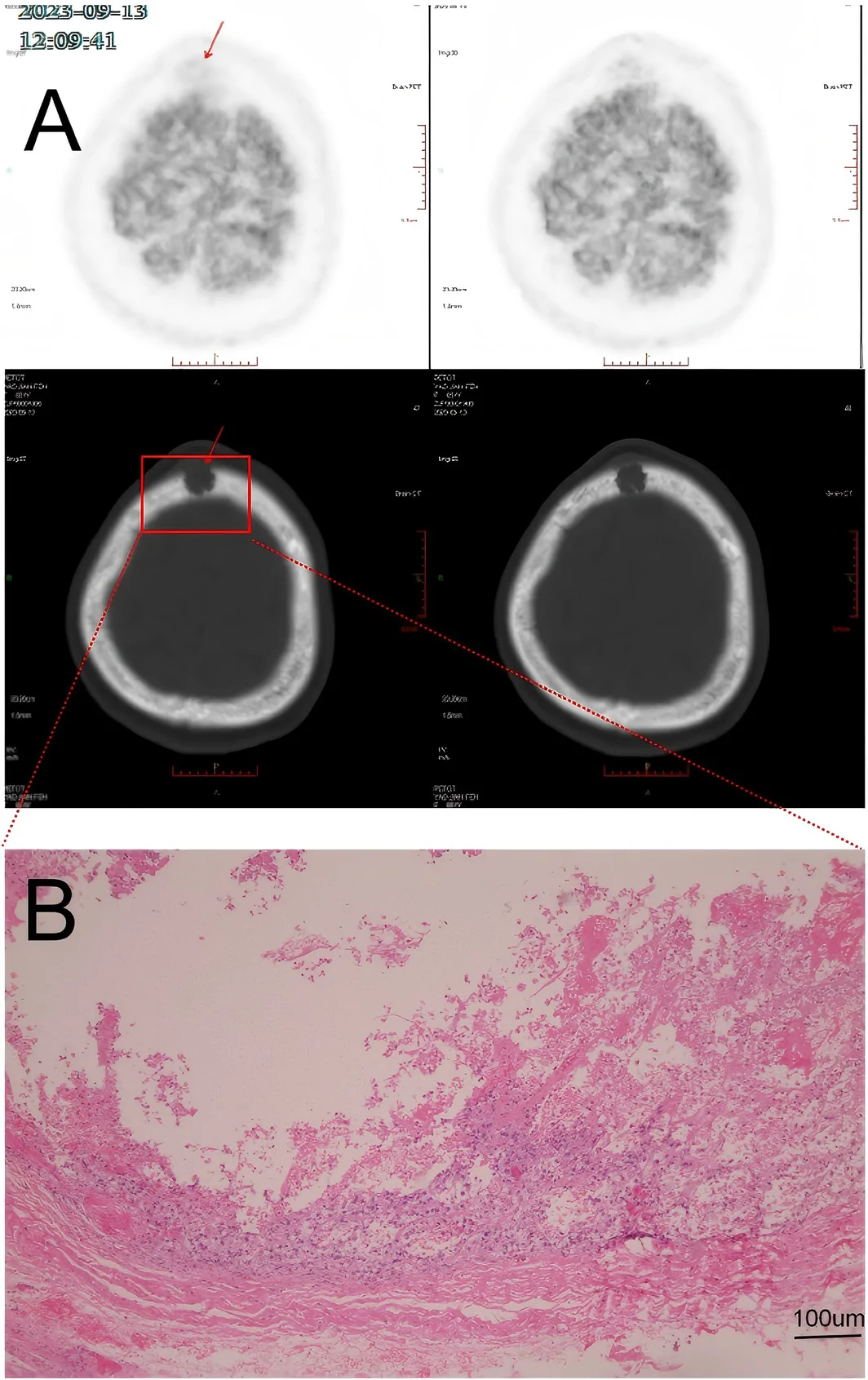
(A) The 18F‐FDG PET/CT demonstrated a metastatic lesion in the right cranial bone, accompanied by H&E‐staining of metastatic lesion (B).

**FIGURE 4:**
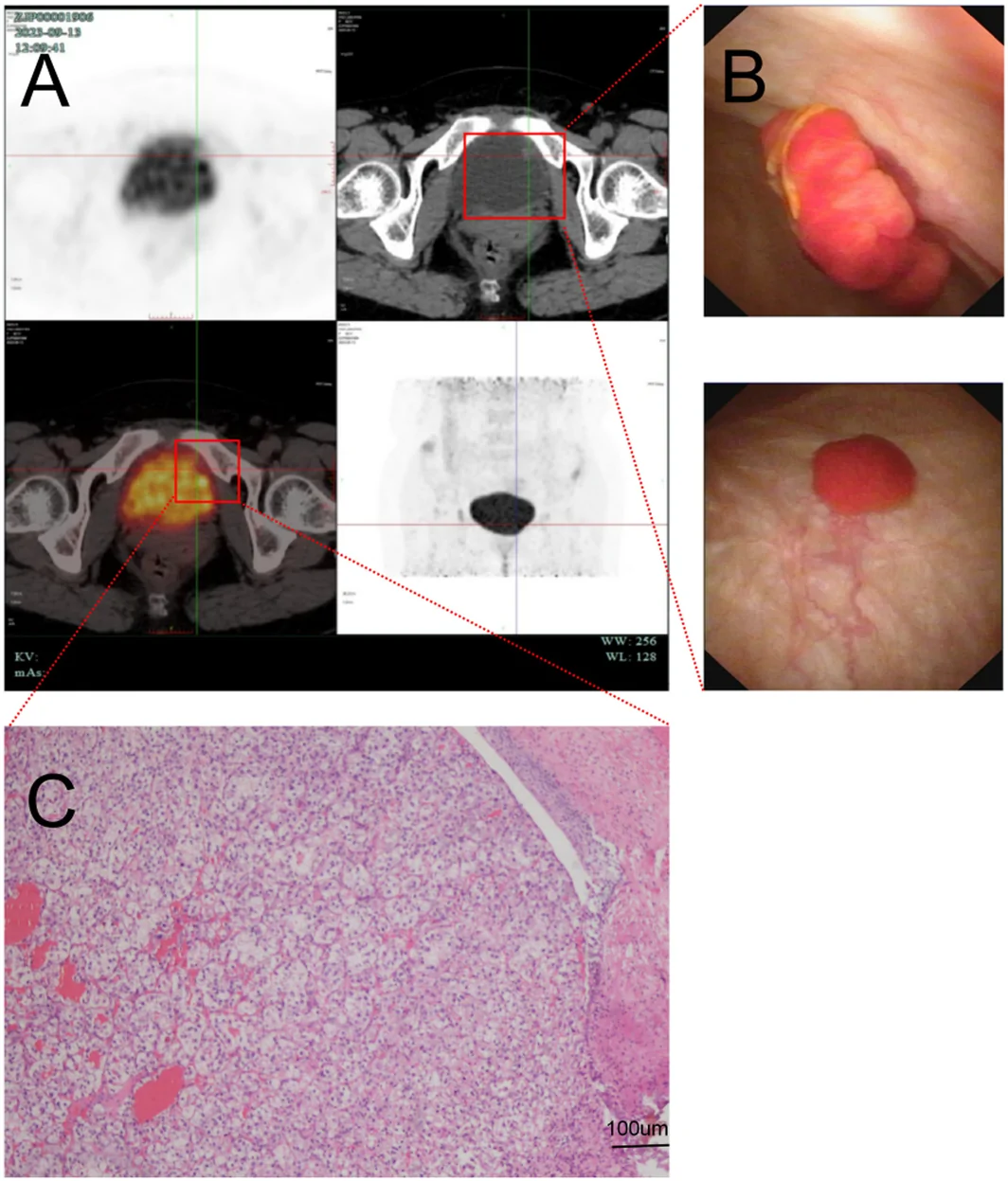
(A) The 18F‐FDG PET/CT demonstrated a metastatic lesion in the bladder, accompanied by cystoscopy (B) and H&E‐staining of the metastatic lesion (C).

**FIGURE 5:**
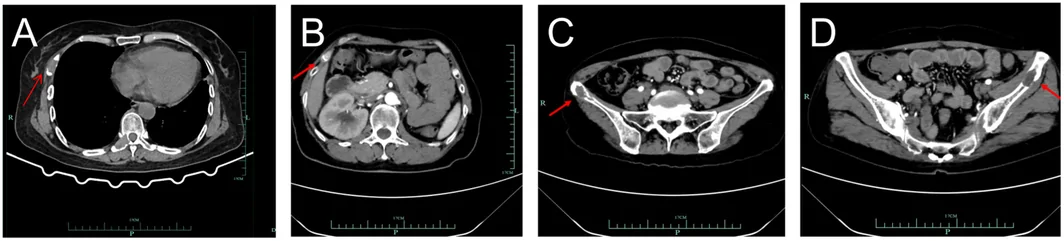
The CT scan revealed multiple metastatic lesions in the ribs (A, B) and pelvis (C, D).

**FIGURE 6:**
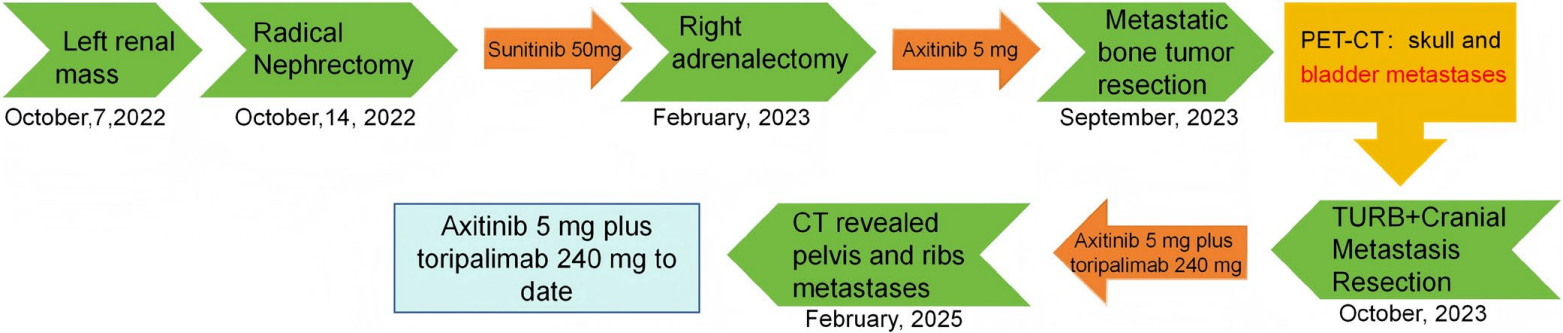
The patient's entire medical course.

This case report describes a 50‐year‐old female patient diagnosed with clear cell renal cell carcinoma (ccRCC) at our hospital, who has remained in generally stable condition during follow‐up but developed systemic metastases including rare bladder involvement. Over 2 years prior, an ultrasound at an external hospital revealed a left renal mass and she was asymptomatic, with no reported hematuria or flank pain. prompting evaluation at our center where contrast‐enhanced CT confirmed left renal carcinoma with ipsilateral adrenal invasion and contralateral adrenal nodules suggestive of metastasis (Figure 1A). After excluding other systemic metastases, the clinical stage was determined as T4N0M1. The patient underwent laparoscopic radical left nephrectomy, with postoperative pathology confirming ccRCC (T4N0M1) (Figure 1C). Sunitinib 50 mg daily as first‐line systemic (targeted) therapy was initiated but discontinued after 3 months due to progression of right adrenal metastasis on follow‐up CT (Figure 1B). Right laparoscopic adrenalectomy revealed ccRCC infiltration (Figure 1D), followed by axitinib 5 mg twice daily with tri‐monthly imaging. The tumor developed adrenal metastasis within 6 months after diagnosis, with rapid disease progression and poor response to TKI therapy. Subsequent follow‐up (beyond 6 months) revealed new metastatic lesions. Ten months post‐surgery, the patient reported right thumb pain, and hand CT showed osteolytic bone destruction at the base of the right first metacarpal with soft tissue involvement, leading to metastatic bone tumor resection with bone grafting and internal fixation (Figure 2A,B). Pathology confirmed ccRCC metastasis (Figure 2C). Systemic F‐18 FDG PET/CT was performed after one week postoperatively and detected skull (Figure 3A) and bladder metastases (Figure 4A), with cystoscopy revealing two red neoplastic lesions confirmed as ccRCC metastasis by transurethral resection pathology and cranial metastasis resection (Figure 4B). Pathology confirmed ccRCC metastasis in skull (Figure 3B) and bladder (Figure 4C). Treatment was switched to axitinib 5 mg twice daily plus toripalimab 240 mg. Three months ago, routine abdominal CT revealed new right ninth rib metastases (Figure 5A,B) and bilateral iliac bone (Figure 5C,D). This case illustrates treatment failure in advanced ccRCC despite 3 months of ongoing targeted/immunotherapy, manifesting as systemic metastases including rare bladder involvement. The patient's entire medical course is illustrated in Figure 6.

## 3 Methods (Differential Diagnosis, Investigations, and Treatment)

The differential diagnosis of renal tumors includes renal tuberculosis, kidney stones, renal cysts, and hamartomas, among others. Abdominal contrast‐enhanced CT results show that the renal mass exhibits significant enhancement during the arterial phase, indicating rich blood supply, with uneven distribution of enhancement suggesting localized ischemic necrosis within the tumor. Therefore, imaging findings suggest the presence of a malignant renal tumor. Radical nephrectomy is the primary treatment for renal tumors. Based on postoperative pathological results, relevant clinical studies, and guidelines, adjuvant targeted therapy is recommended for the patient post‐surgery. During the treatment process, imaging studies, including contrast‐enhanced CT and F18‐PETCT, revealed bone metastasis and bladder metastasis of the tumor. Despite switching targeted drugs and combining immunotherapy, the patient still experienced clinical progression, and pathological findings from localized surgical treatment confirmed metastatic renal cell carcinoma.

## 4 Conclusion and Results(Outcome and Follow‐Up)

This patient initially underwent surgical resection of the affected kidney, which was considered a localized tumor. After adjuvant targeted therapy post‐surgery, the tumor progressed rapidly, leading to metastasis to the contralateral adrenal gland, which was subsequently resected. Following a change in targeted medication, the patient developed localized pain and a bladder mass. After surgical removal of the localized mass, pathology confirmed metastatic renal cell carcinoma. Despite combined immunotherapy, distant tumor progression continued. Although current targeted therapy combined with immunotherapy has significantly improved prognosis and survival in patients with advanced renal cell carcinoma in large prospective clinical studies, some patients remain unresponsive to treatment. This patient is currently under ongoing follow‐up.

## 5 Discussion

This case report describes an asymptomatic patient with advanced RCC detected incidentally during routine physical examination. At diagnosis, contralateral adrenal metastasis was already present. After 3 months of Tyrosine kinase inhibitor (TKI) monotherapy, disease progression was observed in the adrenal metastasis. Subsequently, the patient received combined TKI and immunotherapy. During follow‐up, the patient developed systemic metastases, including a rare bladder metastasis, yet remained free of lower urinary tract symptoms even at this stage. To our knowledge, no previous cases have reported bladder metastasis in advanced RCC following first‐line targeted therapy. A recent literature report documented a case of renal tumor with concurrent bladder metastasis at diagnosis [7]. Previous case reports typically described patients with localized renal tumors who developed isolated bladder metastases several years after undergoing partial or radical nephrectomy, without evidence of metastasis to other sites. Additionally, there have been reports of bladder metastases occurring in patients with non‐classical histological subtypes of renal cell carcinoma [8]. Throughout follow‐up, this patient underwent five salvage tumor resections and has continued combined targeted and immunotherapy to date. Recent follow‐up imaging revealed increased skeletal metastases. TKIs, as first‐line targeted agents for locally advanced and metastatic RCC, can be administered either as monotherapy or in combination with immunotherapy for systemic treatment of advanced RCC patients. Clinical studies have demonstrated that sunitinib significantly prolongs patient survival compared to interferon alpha [9]. However, the patient we reported showed poor response to TKI monotherapy, with disease progression in metastatic lesions. Consequently, we performed salvage surgery to resect the contralateral adrenal metastasis and switched sunitinib to axitinib. Within six months of treatment initiation, imaging revealed disease progression with newly developed bone metastases and bladder metastasis, which were subsequently surgically excised. In light of recent studies demonstrating superior clinical benefits of axitinib combined with toripalimab compared to sunitinib in advanced RCC patients [10], we initiated combination targeted and immunotherapy with the goal of delaying disease progression and improving overall survival. During follow‐up, after one year of continuous combination therapy, the patient developed new bone metastases involving the ribs and pelvis, while cystoscopic examination showed no evidence of bladder tumor recurrence. Based on literature report, 70% of patients with bladder metastasis from renal cell carcinoma present with clinical hematuria [11]. However, in this case, the patient did not exhibit any urinary symptoms but was incidentally diagnosed through imaging. Following transurethral resection of the bladder tumor and combined targeted and immunotherapy, follow‐up examinations showed no signs of recurrent bladder metastasis. Although the patient in this case showed no significant recurrence of bladder tumors after receiving immunotherapy and targeted therapy, multiple bone metastases still developed. Given this challenging clinical course, we present this case to discuss potential subsequent treatment strategies. While current guidelines demonstrate that first‐line therapies can effectively prolong survival and progression‐free survival in advanced RCC, some patients remain treatment‐refractory, developing widespread metastases. For such cases, genomic profiling to identify novel therapeutic targets may offer clinical benefits.

## 6 Conclusion

Bladder metastasis of RCC is relatively rare, and there is currently no standardized clinical management guideline. While resection of bladder metastases may reduce local tumor recurrence, it does not suppress distant metastasis. Targeted therapy and immunotherapy can prolong patient survival. However, clinicians should remain vigilant for potential metastases to other uncommon sites. In this case, the patient's bone metastases continue to progress, indicating a need for further adjustment of the treatment regimen. We look forward to the development of more effective therapies to improve outcomes for advanced RCC patients.

## Author Contributions

**Xiaohong Zheng:** conceptualization, investigation, writing – original draft. **Peng Jiang:** methodology, data curation, supervision. **Jiatong Zhou:** funding acquisition, conceptualization.

## Funding

This work was supported by The National Natural Science Foundation of China (Grant 82504016).

## Ethics Statement

This study was approved by the Medical Ethics Committee at the Institute of The First Affiliated Hospital, Zhejiang University School of Medicine (IIT20260729B).

## Consent

A written informed consent for study participation from this patient was obtained.

## Conflicts of Interest

The authors declare no conflicts of interest.

## Data Availability

The data that support the findings of this study are available from Jiatong Zhou, upon reasonable request.
